# Preparation and Characterization of Aptamers Against *O,p’*-DDT

**DOI:** 10.3390/ijms21062211

**Published:** 2020-03-23

**Authors:** Wei Zhang, Danyang Li, Jianguang Zhang, Lingli Jiang, Zhaofa Li, Jun Sheng Lin

**Affiliations:** School of Medicine, Huaqiao University, Quanzhou 362021, Fujian, China; 17011071007@stu.hqu.edu.cn (W.Z.); lidanyang0805@163.com (D.L.); 1601116007@stu.hqu.edu.cn (J.Z.); 17013071005@stu.hqu.edu.cn (L.J.); lizhaofa@hqu.edu.cn (Z.L.)

**Keywords:** *o,p’*-DDT, aptamer, SELEX, AuNPs, biosensor, real water sample

## Abstract

The compound 1,1,1-trichloro-2-(p-chlorophenyl)-2-(o-chlorophenyl) ethane (*o,p’*-DDT) has been identified as one of the endocrine-disrupting chemicals causing adverse effects on wildlife and even humans through bioaccumulation. Its detection has become increasingly important. We have obtained candidate aptamers binding to *o,p*’-DDT by a systematic evolution of ligands by exponential enrichment (SELEX) protocol. Five out of seventeen candidate sequences were selected for preliminary characterization by SYBR Green I assay. One sequence with highest fluorescence response with *o,p’*-DDT, designated DDT_13, was chosen for further characterization. Its dissociation constant (K_d_) was determined to be 412.3 ± 124.6 nM. DDT_13 exhibited low cross-binding activities on other tested small molecules. The good bioactivities of DDT_13 were demonstrated for the analysis of spiked lake water and tap water samples. This study provides a novel *o,p’*-DDT-specific probe for its future applications.

## 1. Introduction

Dichlorodiphenyltrichloroethane (DDT), a ubiquitous environmental organochlorine contaminant, has been associated with several disorders of the endocrine and reproductive systems in humans and wildlife [1]. Although its usage has been restricted for mosquito control in developing countries with tropical climates, DDT remains active in the environment worldwide and bioaccumulates in the fat stores of animals and humans because of its lipophilic nature and chemical stability [2,3]. It has been postulated that DDT-induced endocrine disruption is caused by the compound binding to sex hormone receptors, which interferes with normal hormone responses [4]. *O,p’*-DDT is the most estrogenic contaminant in commercial DDT (technical grade DDT contains 15–25% of the *o,p’*-DDT) [5]. The results of *in vitro* competitive binding assays have shown that *o,p’*-DDT binds to the estrogen receptor (ER) in mammals, birds, and fish. From *in vivo* studies, *o,p’*-DDT has been found to be estrogenic, as measured by uterotrophic responses in mammals and vitellogenins production in fish [1,6]. *O,p*’-DDT has been identified as a relatively potent estrogen agonist in *in vitro* tests with estrogen-responsive tumor cells and in the yeast estrogen screening (YES) assay [2,7].

The most common methods for *o,p’*-DDT detection are chromatographic-spectroscopic methods (e.g., LC-MS, GC-MS) [8,9]. These techniques are associated with high sensitivity (~pM) but are expensive, sometimes required extensive sample pretreatment (e.g., sample derivatization), are instrumentation- and time-intensive, and cannot be practically used for on-site analysis. Thus, there is an urgent need for the development and innovation of detection system, which should be sensitive, quick, specific, inexpensive and convenient. In general, the antibody-based sensing assays are sensitive enough in practical applications, but antibodies could be degraded when delivered to be used on site. The production of conventional animal-based monoclonal antibodies is tedious, very expensive and challenging.

Aptamers are single-stranded DNA (ssDNA) or RNA, which can fold into 3-dimensional conformations, and bind to user-defined target molecules with very high affinity and specificity. Aptamers are also nucleic acid-based molecular recognition elements that were first introduced independently by three groups in 1990 [10,11,12]. Aptamers can be isolated through an iterative *in vitro* selection process, termed systematic evolution of ligands by exponential enrichment (SELEX). The process begins with the synthesis of a combinatorial random library with almost 10^15^ oligonucleotide sequences and involves repeated steps including selection, separation and amplification. In SELEX, “systematic evolution” means the selection of highly specific ligands (i.e., candidate aptamer) from a random library for a particular target by repeated rounds of target-binding selection, amplification and strand purification [13,14,15]. In brief, the sequences from the initial pool that bind to a target molecule of interest are selected, separated, and amplified by polymerase chain reaction (PCR), thereby enriching candidate sequences for the next selection. ssDNA aptamers have been extensively investigated in biosensing applications because of their low-cost and reversible denaturation [16,17]. Aptamers with conformational changes upon target binding have been reported as highly versatile molecular recognition elements in biosensor development. This study was aimed to isolate ssDNA aptamers with characteristics of conformational changes specifically induced by *o,p’*-DDT-binding. The described strategy of Capture-SELEX matches the aim.

## 2. Results and Discussions

### 2.1. Selection of o,p’-DDT Aptamers

**Figure 1:**
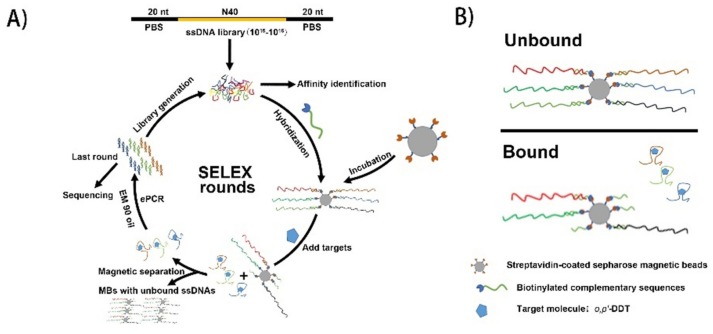
Illustration of the systematic evolution of ligand by exponential enrichment process. (**A**) Library molecules that bind to the target (*o,p’*-DDT) were retrieved and amplified by PCR in one round of SELEX. (**B**) Illustration of the library immobilization process for the modified Capture-SELEX. Biotinylated complementary DNA (cDNA) probes with library ssDNA molecules were first captured on streptavidin coated magnetic beads. Library ssDNA molecules dehybridized by positive target induction were retrieved and subsequently amplified.

The selection of aptamers binding to small molecule targets by SELEX is always a challenge. Small size targets have limited binding sites. Some targets even have only one binding site available, which makes difficulty to immobilize on a solid phase. Immobilization may create steric hindrance also resulting in losing of binding sites before target-ssDNA binding selection. So, the affinity of aptamers to small molecule targets is known to be inferior to large targets such as proteins [18,19]. Capture-SELEX is suitable for the selection of aptamers bound to small molecule targets, because it allows using a free target solution without the loss of possible binding sites and unwanted steric restrictions [20]. In this study, the Capture-SELEX (Figure 1) was designed to isolate the sequences who changed their conformations due to binding with *o,p’*-DDT thus dissociated from their complementary DNA (cDNA) fragments, therefore transferred from the solid (magnetic beads) to liquid phase (binding buffer). In brief, the ssDNA oligonucleotides of library were captured by cDNA fragments designated Lib3-F-CS-Biotin, which were coated on magnetic beads in binding buffer. *O,p’*-DDT was applied to the system to induce the release of target-bound therefore conformation-changed oligonucleotides from the complementary fragments. The *o,p’*-DDT-bound oligonucleotides were then selected and amplified as a sub-library for next round of *in vitro* selection (Figure 1). SELEX selection generally has to undergo multiple rounds to enrich candidate sequence species. It is very important to monitor whether the specific candidates are enriched after selection step in the SELEX process. In this study, we employed two types of PCR techniques, qPCR and emulsion PCR (ePCR). We conducted qPCR to monitor the enrichment of libraries after each selection step. Results showed that the amplification curve of round 13 was increasing steeply, when compared to that of other rounds (Supplementary Figure S1). These results were in accordance with a previous report used for monitoring the selection progress of aptamer [21]. The main disadvantage of conventional PCR amplification is that overamplification increase nonspecific hybridization among different products and by-products, which may cause the loss of potential specific aptamers, inefficient selection, and even selection failure. It has been reported that ePCR could overcome the shortcoming of conventional PCR. During ePCR, different templates are separated by emulsion particles, allowing single-molecule PCR, and avoiding nonspecific hybridization [22].

### 2.2. Characterization and Analysis of the Last Round Library and the Candidate Aptamers

**Figure 2:**
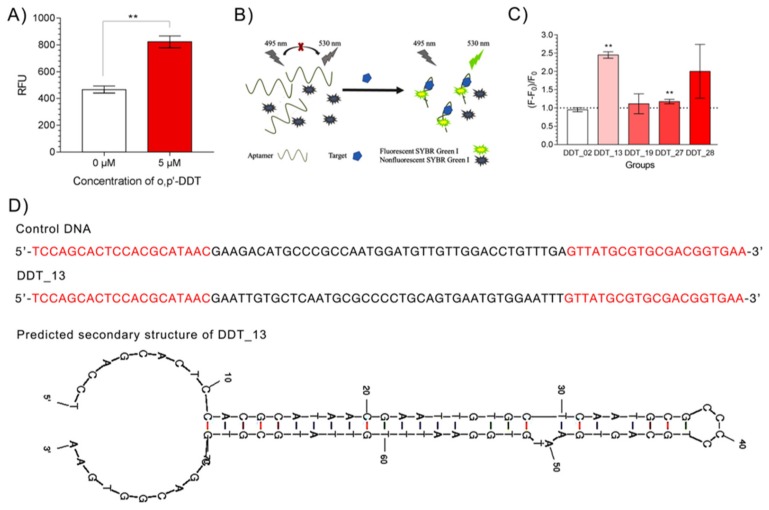
Characterization and analysis of the last round library and the candidate aptamers. (**A**) The fluorescence intensity of the library in round 13 against *o,p’*-DDT. (**B**) The principle of SYBR Green I (SGI) assay. (**C**) Fluorescence responses of five candidate sequences by SYBR Green I (SGI) assay. (**D**) Comparison of DDT_13 and control sequence. Full sequence of the control DNA and the *o,p’*-DDT candidate aptamer, DDT_13 were presented. The red letters are primer binding sites. Secondary structure of DDT_13 was predicted by Mfold software. ** means *p* value < 0.01, when compared with that of the binding buffer group.

Thirteenth rounds of SELEX were performed targeting *o,p’*-DDT. The FAM-labeled sub-library derived from round 13 was immobilized on the magnetic beads and incubated with or without *o,p’*-DDT in the buffer. The releasing library molecules were detected by fluorescence spectrophotometer respectively. Results showed that the fluorescence response against *o,p’*-DDT was significantly higher than that of control group (Figure 2A). Then the library was prepared for cloning and sequencing. Seventeen clones were sequenced and analyzed (Supplementary Table S1). Although no obvious conservative motif was identified, we observed that five of the sequences, which were predicted with lower dG values. In general, a lower dG value usually indicates that the sequence might be folded up with more stable secondary structure(s), which help to maintain the stability of the target/aptamer complexes [23,24]. As a result, candidate aptamers (DDT_02, DDT_13, DDT_19, DDT_27, and DDT_28) with lower dG value were choose for further analysis. The binding affinity between the selected candidate aptamers and *o,p’*-DDT was characterized by using SYBR Green I (SGI) assay as previously described with slight modification [25]. SGI assay is a simple assay, recommended for the screening of small molecule aptamers [26]. SGI is a green fluorescence dye specific for double-stranded DNA by means of DNA intercalation. The dye itself does not have a fluorescence signal and is commonly used in real time quantitative PCR (RT-qPCR) for template amplification monitoring [27]. Upon the aptamer molecules bound to their targets, their conformation changed leading more or less regions to become double-stranded and fluorescence emission increased or decrease (Figure 2B). Results showed that ratio of fluorescence response of DDT_13 was significantly higher than that of DDT_02, DDT_19, and DDT_27 (Figure 2C). Though the mean of ratio of fluorescence of DDT_28 was higher than that of DDT_02, DDT_19 and DDT_27, the large variation (large standard deviation) in replicated assays indicated that the signals of DDT_28 were not stable enough (Figure 2C). These results indicated that the designated DDT_13 with the highest fluorescence response can be chosen for further analysis. Mfold predicted that one of the secondary structures of the DDT_13 (Figure 2D) contained one long hairpin structure and a small second hairpin (dG = −42.42 kcal/mol).

In this study, five of seventeen sequences, which were predicted with binding potential with *o,p’*-DDT, were selected by the strategy with lower dG values. Results of SGI assays showed that fluorescence responses of three of the selected sequences (DDT_02, DDT_19, and DDT_27) were weakly activated by *o,p’*-DDT (Figure 2C). The difference between experimental and predicted results revealed that unselected sequences are also valuable for further investigation.

### 2.3. Binding Tests

**Figure 3:**
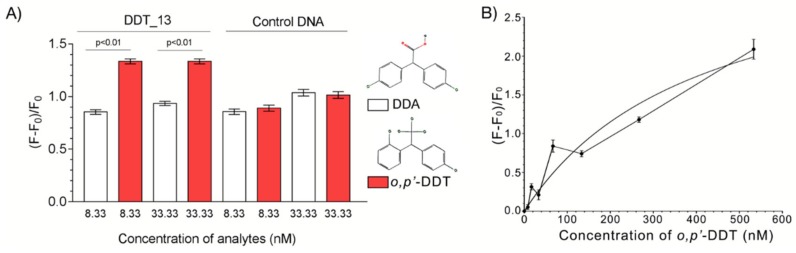
Binding tests between DDT_13 and its target *o,p’*-DDT. (**A**) Fluorescence response to the DDT_13 and control DNA while adding with *o,p’*-DDT and a interfering homologue molecule DDA; Insert images: the 2D structure of *o,p’*-DDT and DDA; (**B**) Determination of the dissociation constant (Kd) of DDT_13.

All the aptamers isolated by the Capture-SELEX protocol are intended to demonstrate the feature of structure-switching with the target, and other non-switching aptamers are expected to be stayed with the solid phase and couldn’t be included, during the partition step of each selection cycle [25]. Thus, these structure-switching aptamers can induce a fluorescence response while bound to their target. The binding affinity between the DDT_13 and *o,p’*-DDT was characterized by using SGI assay as previously described. Compared with the interfering homologue molecule bis (4-chlorophenyl) acetic acid (DDA), the control DNA did not induce the enhancement of fluorescence signals, while DDT_13 significantly enhanced the fluorescence signals (Figure 3A). These results indicate that DDT_13 can change its 3D structure upon binding to the target of *o,p’*-DDT, and this alteration can be detected by the change of fluorescence intensity. The dissociation constant (Kd) of DDT_13 was analyzed, and was calculated as 412.3 ± 124.6 nM (Figure 3B). This value is good enough for small molecule aptamers [18,19].

**Figure 4:**
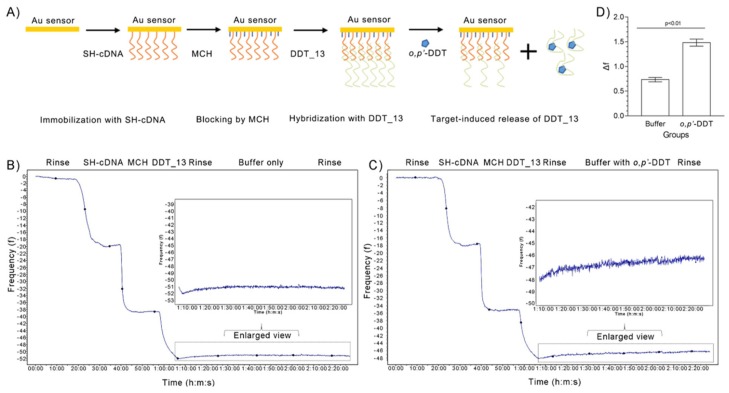
Binding tests by quartz crystal microbalance (QCM) assay between DDT_13 and its target *o,p’*-DDT. (**A**) Detection principle of QCM. (**B**, **C**) QCM analysis of binding affinity of DDT_13 without (**B**) and with its target *o,p’*-DDT (**C**) in binding buffer. (**D**) The changes of the f value (Δf) of the sensor crystal after binding without and with *o,p’*-DDT, respectively.

To further confirm the binding activity between *o,p’*-DDT and DDT_13, the changes of DDT_13 binding to various amounts of *o,p’*-DDT (0 and 0.5 μM) were studied by quartz crystal microbalance (QCM), a sensitive and versatile tool for measuring adsorption of various compounds to its surfaces, e.g., small molecules [28]. In this assay, the thiol-capture DNA (SH-cDNA) was firstly immobilized onto the Au sensors by means of Au–SH interaction. Then the DDT_13 was introduced to the system by hybridization with SH-cDNA. Similar as the SELEX process, once the *o,p’*-DDT bound with DDT_13, the DDT_13 would release from the sensor. The mass change caused by releasing of DDT_13 molecule can be easily detected by QCM (Figure 4A). Results showed that changes of the f value (Δf) of the sensor crystal occurred after binding to *o,p’*-DDT molecules (Figure 4B–D). This suggests that the binding of *o,p’*-DDT to DDT_13 embedded form led to the reduction of DNA base accumulation. Therefore, DDT_13 conformation changed after binding with *o,p’*-DDT, which confirmed the interaction between DDT_13 and *o,p’*-DDT.

### 2.4. Gold Nanoparticles (AuNPs) Assay

**Figure 5:**
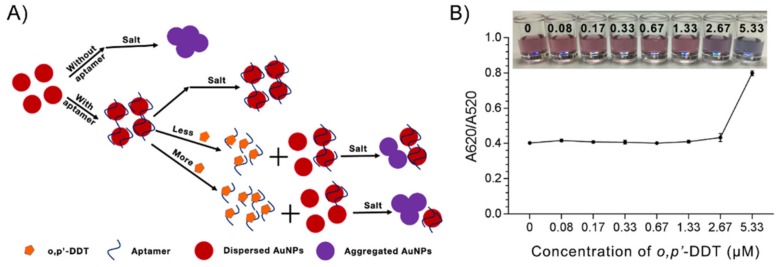
Gold Nanoparticles (AuNPs) colorimetric assay. (**A**) Schematic principle of the assay. (**B**) Aggregation response to *o,p’*-DDT addition quantified by absorbance ratio. Insert image: Digital image of the samples represented in (**B**).

AuNPs colorimetric assay has been commonly adopted as a rapid detection platform for aptamer−target binding [29]. In brief, the assay principle relies on the pink-red to blue-purple shift observed in AuNPs aggregation. Many studies showed ssDNA aptamers coated AuNPs resisted salt induced aggregation, the blue-purple color shift was not observed [30]. Upon the addition of the target, ssDNA aptamer detached from AuNPs, the salt induced blue-purple color shift was observed (Figure 5A). This change in color could be quantified with absorbance measurement at 520 and 620 nm. Our results showed that salt induced AuNPs aggregation was observed when the increasing concentrations of *o,p’*-DDT were added to DDT_13 coated AuNPs (Figure 5B).

### 2.5. The Specificity

**Figure 6:**
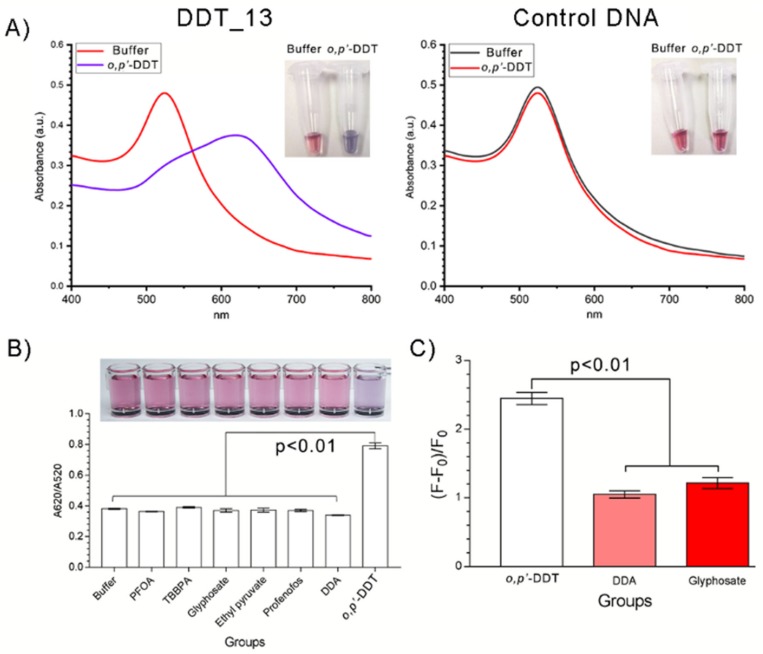
Analysis of selectivity of DDT_13. (**A**) The signal changing induced by control DNA or the DDT_13; (**B**) Signal changing of DDT_13 specifically responded to *o,p’*-DDT but not to other kinds of small molecules; (**C**) Fluorescence response to *o,p’*-DDT and other small molecules.

We conducted both AuNPs assay and SGI assay for specific analysis (Figure 4). In AuNPs assay, DDT_13 did, while the control DNA did not, induce the color changing of AuNPs (Figure 6A). We employed UV-vis absorption spectrum to characterize the aggregation behavior of AuNPs and confirm the principle of the AuNPs assay [31]. Results showed that the AuNPs distribution was different when treated with various substances (Figure 6A). In DDT_13 group, the AuNPs were well dispersed in the buffer group with an absorption peak at 520 nm, which was consistent with the original AuNPs. When added with *o,p’*-DDT, significant aggregation of AuNPs was observed due to the separation of DDT_13 from the AuNPs by the formation of DDT_13/*o,p’*-DDT complexes. Therefore, the absorption peak was changed from 520 nm to 620 nm following the aggregation of AuNPs. Similarly, the color of the solution was changed from red to blue-violet, as shown in Figure 6A. In the control DNA group, there was no significant changes in both the absorption peak and visible color after adding with *o,p’*-DDT (Figure 6A).

For environmental pollution, a variety of pollutants coexisted in a complex environment such as water. Thus, the specificity of recognition module is very important for biosensor development. *O,p’*-DDT, an estrogen mimic, is identified as an endocrine-disrupting chemicals (EDCs) [32]. Here we selected three EDCs (Pentadecafluorooctanoic acid (PFOA), Tetrabromobisphenol A (TBBPA), and Ethyl pyruvate), two pesticide residues (Glyphosate and Profenofos), and one molecule of DDA as the interferents of this system whether it could attain high selectivity analysis. Among these small molecular interferents, DDA is a homologue of *o,p’*-DDT. Results of AuNPs assay showed that the response signal of DDT_13 binding with *o,p’*-DDT was exceedingly higher than other small molecules, which indicated that DDT_13 had high specificity (Figure 6B). In more, results of SGI assay showed that the fluorescence response of *o,p’*-DDT was significantly higher than that of other small molecules (Figure 6C). Thus, our cross-selectivity studies showed that DDT_13 exhibits a good selectivity to *o,p’*-DDT in comparison to control DNA and other interfering molecules.

### 2.6. Tests in Water

**Figure 7:**
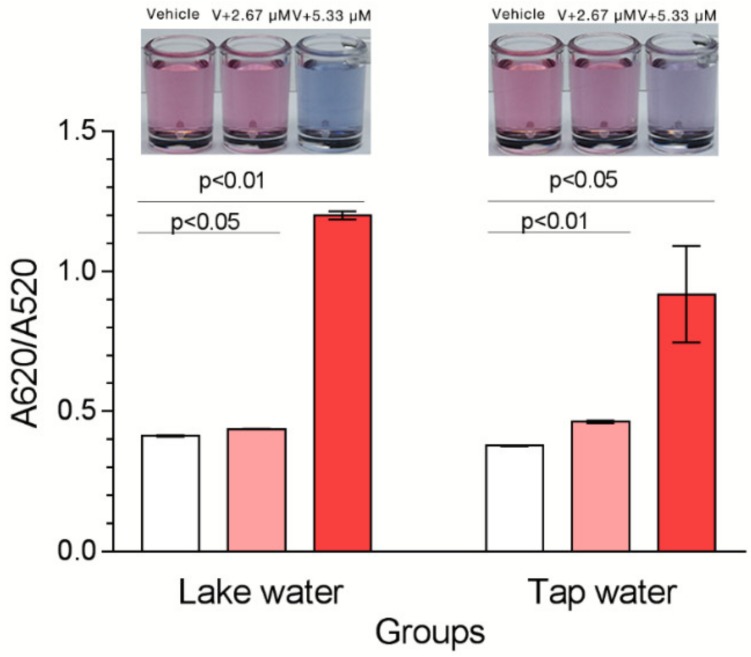
Gold Nanoparticles (AuNPs) assay in water samples. Signal changing of DDT_13 responses in water samples. Insert images: the color changing induced by the DDT_13.

*O,p’*-DDT is usually analyzed by hardware-equipped and time-consuming chromatographic methods [8,9]. To demonstrate the feasibility of DDT_13 in the detection of *o,p’*-DDT in real water, we tested it in actual samples with different concentrations of *o,p’*-DDT. Results showed that ratios of A620/A520 of water samples containing *o,p’*-DDTs were higher than of the negative controls (Figure 7). Furthermore, the signal changing of lake water is relative higher than that of tap water. Water samples with different molecules such as saline ions can cause different signal responses.

In humans, a survey of 200 mothers showed that *o,p′*-DDT in maternal serum was detected in 82.5% of samples at median concentration of 0.22 ng/mL [33]. This study indicates a possible impact of prenatal exposure to *o,p’*-DDT on newborn anthropometric measurements. Another study reported that the prenatal exposure (median concentration of 1.2 ng/g lipid) during pregnancy was associated with several adiposity measures in boys [34]. In wildlife, *in vivo* studies have shown that *o,p′*-DDT can induce intersex in Japanese medaka at much higher exposure concentrations (5−50 μg/L) [35,36]. These studies indicate that the potential toxic concentration of *o,p’*-DDT for wildlife and humans are at ng/mL level (sub-micro mole level). Our studies showed that the novel specific aptamer DDT_13 had the high affinity and specificity against *o,p’*-DDT. Both SGI assay (nano mole level) and AuNPs assay (micro mole level) showed that DDT_13 has the potential for detection of *o,p’*-DDT. With the further development of various signal amplification strategies, the DDT_13-based biosensor will contribute to detection of *o,p’*-DDT in real samples for humans and wildlife.

## 3. Conclusions

This is the first study to identify a new aptamer that binds to *o,p’*-DDT with high affinity and specificity. In this study, we conducted a Capture-SELEX protocol to isolate *o,p’*-DDT specific aptamers. After 13 rounds of selection process, DDT_13 was isolated and characterized. Its dissociation constant (K_d_) was determined as 412.3±124.6 nM. It showed low cross-selectivity to other small interfering molecules. The results from two spiked real water samples demonstrated that DDT_13 has potential in the detection of *o,p’*-DDT in real water samples. To our knowledge, neither aptamer nor any aptasensor for the detection of *o,p’*-DDT has yet been reported.

## 4. Materials and Methods

### 4.1. Chemicals and Reagents

The analytical standard *o,p’*-DDT and HAuCl_4_·4H_2_O were purchased from Aladdin Co., Ltd. (Shanghai, China). 2,2-bis (4-chlorophenyl) acetic acid (DDA) (>98%), triton X-100 and mineral oil were purchased from Sigma (Shanghai, China). Pfu DNA polymerase was purchased from Sangon Biotech Ltd. (Shanghai, China). Taq DNA polymerase and dNTPs were purchased from TAKARA Ltd. (Dalian, China). SYBR Green I (10,000 ×) was purchased from Solarbio Science & Technology Co., Ltd. (Beijing, China). Streptavidin Mag Sepharose beads were purchased from Enriching Ltd. (Shanghai, China). EvaGreen was purchased from Shanghai Open Biotechnology Ltd. (Shanghai, China). EM 90 oil was purchased from ABIL^®^ (Essen, Germany). All other analytical pure chemical reagents were purchased from Sinopharm Chemical Reagents Ltd. (Shanghai, China). The quartz crystals coated with gold (d Au = 100 nm) (QSX301) with a fundamental resonance frequency of 5 MHz were purchased from Q-Sense (Biolin Scientific AB/Q-Sense, Gothenburg, Sweden) and used as received for the QCM-D measurements. Sodium dodecyl sulfate (SDS, 99% purity, Sigma-Aldrich Co., Munich, Germany) was used to clean the crystals, QCM-D chamber, and connecting tubes. Other solutions used in the experiments were treated with sterilization Elix water. The aptamer library was synthesized by Sangon Biotech Ltd. (Shanghai, China). The primers and aptamers were synthesized by Genscript Biotechnology Ltd. (Nanjing, China). Secondary structures were calculated with Mfold (Missouri, USA) online bioinformatics platforms (http://unafold.rna.albany.edu/?q=mfold/DNA-Folding-Form). All chemicals were used as received.

### 4.2. Apparatus

Quartz Crystal Microbalance with Dissipation Monitoring (QCM-D, E4) (Biolin Scientific AB/Q-Sense, Gothenburg, Sweden) was used for affinity analysis; Real-time quantitative PCR system (TL980) (Xi’an Tianlong Science and Technology Ltd., Xi,an, China) was applied for monitoring the richness of library; PCR (T960) (Heal Force Bio-meditech Holdings Limited, Shanghai, China) was used for amplifying; F-7000 fluorescence spectrophotometer (Hitachi, Tokyo, Japan) was used for fluorescence detection; Microplate reader (Flash spectrum, Shanghai, China) was used for recording the absorbance; Micronucleic acid quantifier 3000 (Hangzhou Allsheng Instruments Co, Ltd., Hangzhou, China) was applied for ssDNA quantitation.

### 4.3. In Vitro Selection for o,p’-DDT-Specific ssDNA Aptamers

The selection process was performed as previously described with modifications [37]. In brief, in the first round, the synthetic ssDNA oligonucleotide library (library Lib3_bank, Table 1, 1 OD) was dissolved in binding buffer (1mM CaCl_2_, 2.5 mM KCl, 1.5 mM KH_2_PO_4_, 0.5 mM MgCl_2_·6H_2_O, 137 mM NaCl, 8mM Na_2_HPO_4_, pH 7.2). Lib3-F-CS-Biotin (Table 1) was mixed with the library at a ratio of 2:1(mole ratio), and slowly denatured and renatured (95 ° C for 10 min, 60 °C for 1 min, 25 °C for 1 min). The above mixture was added to the 400 μL streptavidin magnetic beads (10 mg/mL) and incubated (the magnetic beads were washed 6 times with the binding buffer before use) for 45 min. The magnetic beads were washed 6 times, and incubated for 90 min with *o,p’*-DDT in binding buffer (200 μL) to a final concentration of 10 μM at room temperature. The supernatant was collected by magnetic separation, monitored by real-time quantitative PCR (qPCR) and amplified by emulsion polymerase chain reaction (ePCR) with EM 90 oil (50 mL EM 90 oil with 1 mL EM 90, 25 μL triton X-100, and 49 mL mineral oil).

The PCR product was concentrated with n-butyl alcohol, then it was mixed with 2× TBE-urea buffer and boiled for 10 min. The sample was separated by 8% denatured polyacrylamide gel electrophoresis (PAGE) (300 V, 18 min). The fluorescent strip was cut off and boiled to separate the secondary ssDNA library, which was dialyzed overnight in binding buffer at 4 °C. After measuring of the secondary library concentration, the next round of selection was conducted (Figure 1). The following rounds of SELEX were subsequently conducted following the same procedure described in round 1 except that the ~14 pmol library was input to increase the selection stringency.

### 4.4. Enrichment Monitoring

qPCR method was established for monitoring the selection process. In brief, 2 μL of the template was mixed with qPCR mix (Lib3-F/R, Table 1), and the qPCR was performed following the program: 95 °C for 5 min, 95 °C for 0.5 min, 60 min at 0.5 °C, and 72 °C for 0.5 min with 26 cycles. The enriched libraries (eluting buffer) from each round and the washing buffers were monitored.

### 4.5. Determination of the Fluorescence Intensity Induced by the Last Round Library

The FAM-labeled library of round 13 (700 nM in 100 μL) was hybrided with Lib3-F-CS-Biotin (Table 1). The above mixture was added to 70 μL suspension of streptavidin magnetic beads and incubated for 45 min. The magnetic beads were washed 6 times, and incubated for 90 min with *o,p’*-DDT (final concentration at 5 μM) in binding buffer or binding buffer only at room temperature. The fluorescence (excitation at 480 nm, emission at 520 nm) was recorded using the F-7000 fluorescence spectrophotometer (Hitachi, Tokyo, Japan).

### 4.6. Cloning and Sequencing of the o,p’-DDT-Binding ssDNA Aptamers

After the final SELEX round, the library with determined affinity was amplified using taq DNA polymerase with the non-modified primers (Lib3-F/R, Table 1). The dsDNA product was purified with a DNA purification kit (Sangon, Shanghai, China) and cloned using a pEASY^®^-Blunt3 Cloning Kit (TransGen, Beijing, China). Escherichia coli DH5α strain rubidium competent cells were transformed with the ligation mixture. Numbering clones were picked out and identified by PCR with common vector primers (Supplementary Figure S2). The inserts were Sanger sequenced by Sangon Ltd. (Shanghai, China).

### 4.7. Binding Tests of Active Aptamers

In brief, the assay was performed as previously described with slight modifications [25]. To test the binding ability of aptamer candidates, a premix containing 1.5 μM of the aptamer in binding buffer was prepared for each oligonucleotide. The premix was pipetted by 70 µL to the 1.5 mL centrifuge tube containing 70 µL different *o,p’*-DDT (500 nM in final for tests of candidate aptamers) or DDA solution, each in triplicates. The mixture was incubated for 45 min, at room temperature, and then 70 µL of the 1.5× SYBR Green I (SGI) solution in binding buffer, was added to each well. After 2 h of incubation, the fluorescence of the SGI (excitation at 495 nm, emission at 525 nm) was recorded using the F-7000 fluorescence spectrophotometer (Hitachi, Tokyo, Japan). All fluorescence readings were normalized with ((F − F_0_)/F_0_), where F_0_ was the fluorescence reading from the binding buffer control. Kd was calculated by fitting the binding data to a one-site saturation equation (Y = (F − F_0_) × X/(Kd + X), where F is the sample fluorescence, F_0_ is fluorescence of the probe without o,p’-DDT, in the GraphPad Prism 7 software (version 7.00, California, USA) (www.graphpad.com). To determine the specificity of the aptamer. A similar experiment was performed, where the interfering substances (DDA and Glyphosate) used in the detection were prepared at 5 μM. Samples were prepared in triplicate for assays.

### 4.8. The Quartz Crystal Microbalance (QCM) Analysis

The QCM technique is based on a quartz crystal sandwiched between two gold electrodes [38]. QCM-D measurements were performed using QSense E4 instruments (Biolin Scientific, Gothenburg, Sweden) equipped with four flow modules. Prior to use, the sensors (gold-coating, d Au = 100 nm) were exposed to a UV-ozone treatment for 10 min and then immersed in washing buffer (H_2_O:H_2_O_2_:NH_3_·H_2_O = 10:2:2) under stirring for 10 min (70 °C).

The measurement of bound mass is provided by changes in the resonance frequency, f, of the sensor crystal. Here, f was measured at the fundamental resonance frequency (4.95 MHz) as well as at the third, fifth, seventh, ninth, eleventh and thirteen overtones (i = 3, 5, 7, 9, 11, and 13). Experiments were conducted in a continuous flow of buffer with a flow rate of 50 μL.min^-1^ by using a peristaltic pump (ISM935C, Ismatec, Wertheim, Germany). The temperature of the E4 QCM-D platform and all solutions were stabilized to ensure stable operation at 25 °C. All buffers were previously degassed in order to avoid bubble formation in the fluidic system.

In brief, the thiol-capture DNA (SH-cDNA, 0.5 μM of 500 μL) was firstly coupled onto the sensors by using Au-SH interaction. Then the DDT_13 (0.5 μM of 500 μL) was injected and hybrided with the cDNA after blocking with mercapto-1-hexanol (MCH, 1 mM of 500 μL). The buffer without and with *o,p’*-DDT (0.5 μM) was pumped into the system for affinity measurement, respectively. Once the target binding to the aptamer, the aptamer release from the cDNA by structure changing. The change of mass can be easily detected by QCM. Δf*_o,p’_*_-DDT_ and Δf_buffer_ was calculated by the change of f value before and after pumping the buffer with and without *o,p’*-DDT, respectively.

### 4.9. Gold Nanoparticles (AuNPs)-based Colorimetric Assay

AuNPs were prepared as previous report by reducing chloroauric acid with sodium citrate [39]. The concentration of AuNPs in ultrapure water was determined to be 8.76 nM using UV-vis spectroscopy according to the previously reported method [40]. The assay was performed as previously described with optimizations [37]. In brief, 50 μL of aptamer at 0.48 μM and 50 μL of AuNPs from the stock were incubated for 45 min at room temperature. Targets at varying concentrations in 50 μL total volume were added to the aptamer-gold mixture and incubated for 45 min at room temperature. Lastly, 10 μL of 1.2 M sodium chloride (NaCl) solution was added to each mixture for 20 min. The aggregation pattern was observed by naked eye, and in addition transferred to a clear 96-well plate for digital imaging and absorbance scan at 520 and 620 nm with microplate reader (Flash spectrum, Shanghai, China).

In order to determine the specificity of the aptamer, a similar experiment was performed, where all interfering substances (DDA, PFOA, TBBPA, Glyphosate, Ethyl pyruvate, and Profenofos) used in the detection were prepared at 16 μM (TBBPA was at 7.4 μM). Samples were prepared in triplicate for assays.

### 4.10. Functional Studies in Water Samples

The water samples were collected from tap water (Quanzhou, China) and lake water (Qiuzhong lake in Huaqiao University, Quanzhou, China). Then the filter paper and 0.22 μm membrane were consecutively employed from samples purification [41]. All the samples were collected in May 2018, stored at 4°C. The preprocessed samples spiked with *o,p’*-DDT were detected by as prepared colorimetric method illustrated above. Water samples without adding *o,p’*-DDT were introduced as negative controls.

### 4.11. Statistical Analysis

All experiments in this study were performed at least in triplicate for each control and treatment group. The numeric data are expressed as the mean ± SD. Differences between groups were evaluated using Student’s t test. *p* < 0.05 was considered statistically significant. GraphPad Prism 7 (version 7.00, California, USA) (www.graphpad.com) was used to display and analyze the data.

## Figures and Tables

**Table 1:** Library and primers information.

Name	Sequence (5′–3′)	Description
library Lib3_bank	TCCAGCACTCCACGCATAAC(N40)GTTATGCGTGCGACGGTGAA	Initial DNA pool used in SELEX
Lib3-F-CS-Biotin	GTTATGCGTGGAGTGCTGGA	3′ modified with biotin for capturing ssDNA in SELEX
Thiol-capture DNA (SH-cDNA)	GTTATGCGTGGAGTGCTGGA	3′ modified with SH-SH for capturing candidate aptamers in QCM
Lib3-F	TCCAGCACTCCACGCATAAC	Forward primer used for qPCR in SELEX
Lib3-R	TTCACCGTCGCACGCATAAC	Reverse primer used for qPCR in SELEX
Lib3-F-FAM	TCCAGCACTCCACGCATAAC	5′ modified with 6-carboxyfluorescein (FAM) forward primer for preparing sub-libraries in SELEX
Lib3-R-ployA	AAAAAAAAAAAAAAAAAAAAAAAAA-Spacer 18- TTCACCGTCGCACGCATAAC	5′modified with ploy A with spacer 18 reverse primer for preparing sub-libraries in SELEX

## Supplementary Materials

The following are available online at https://www.mdpi.com/1422-0067/21/6/2211/s1, Figure S1 Amplification curve of qPCR for each round of selection. Figure S2 Identification of positive clones by electrophoresis. Arrows mean the clones with insertion of PCR products. Table S1 Seventeen of post round 13 library clones were sequenced and analyzed for consensus sequence family.
