# Supplementary material for: Preparation and Characterization of Aptamers Against O,p’-DDT
Source: Int J Mol Sci. 2020 Mar 23;21(6):2211. doi: 10.3390/ijms21062211 (PMC7139375; doi:10.3390/ijms21062211)
Supplement: Supplementary file 1 [file ijms-21-02211-s001.pdf]

International Journal of
Molecular Sciences

MDPI

Supplementary Materials

1. Figures legends

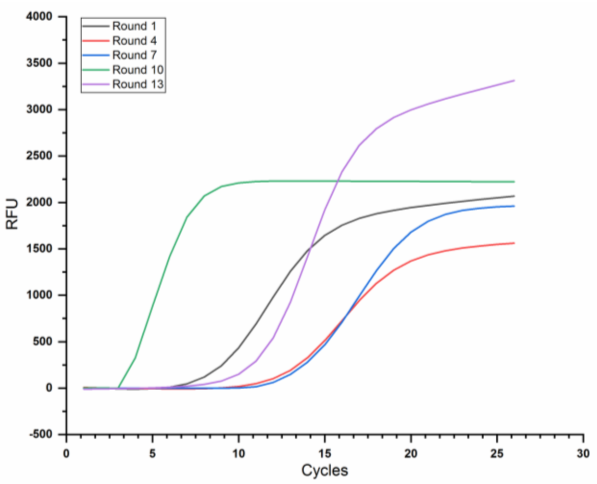

Figure S1 Amplification curve of qPCR for each round of selection.

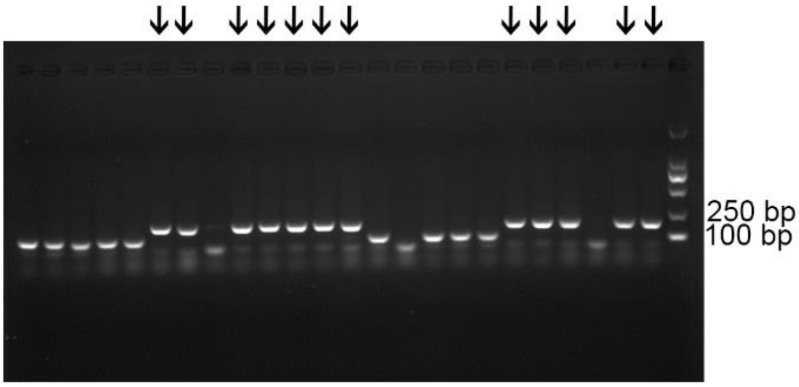

Figure S2 Identification of positive clones by electrophoresis. Arrows mean the clones with right insertions.

International Journal of
Molecular Sciences

2. Table legend

Table S1 Seventeen of post round 13 library clones were sequenced and analyzed for consensus sequence family

Item	Sequence (5′-3′)	dG (kcal/mol)
DDT_02	TCCAGCACTCCACGCATAACAGTTCGAGGCAACAAGGCTGATCAGTCTGATGTTTGAAATGTTATGCGTGCGACGGTGAA	−41.3
DDT_03	TCCAGCACTCCACGCATAACCGTCCACTGCTCGAATACAGCTTCGTCTCTTGCCAAGTGTGTTATGCGTGCGACGGTGAA	−34.71
DDT_06	TCCAGCACTCCACGCATAACCGCGCCACTCCAGAGAGTACATTCCAGTTCGGCCACATTTGGTTATGCGTGCGACGGTGAA	−36.50
DDT_08	TCCAGCACTCCACGCATAACCCACCACCGACAATAACCTTCGCGGAACAAACTGGGGTCTCGTTATGCGTGCGACGGTGAA	−35.97
DDT_10	TCCAGCACTCCACGCATAACCCAGGCGCACACTCGTTCCACTCTCAGTTAAGCAAACCGTGTTATGCGTGCGACGGTGAA	−32.15
DDT_13	TCCAGCACTCCACGCATAACGAATTGTGCTCAATGCGCCCCTGCAGTGAATGTGGAATTTGTTATGCGTGCGACGGTGAA	−42.42
DDT_14	TCCAGCACTCCACGCATAACCACCCCACGTAGTGCCATAAAGAATTGGTCATCGTTTCCCCGTTATGCGTGCGACGGTGAA	−34.01
DDT_16	TCCAGCACTCCACGCATAACCACGCCGTCCGTCATACACGCTCCGTCAACACGTCCTTCTGGTTATGCGTGCGACGGTGAA	−36.57
DDT_18	TCCAGCACTCCACGCATAACCGCCGTACTTACTACTCTGCCACCAAATACATGCACTACCCGTTATGCGTGCGACGGTGAA	−29.32
DDT_19	TCCAGCACTCCACGCATAACCGACCAACTGTCTCGTGGTTGGTTCCGACTACTCAGCTCGGTTATGCGTGCGACGGTGAA	−41.04
DDT_23	TCCAGCACTCCACGCATAACCTGGCAACAACATTCGCTACGACCTGAAGCTCCTGACCTTTGTTATGCGTGCGACGGTGAA	−34.38
DDT_24	TCCAGCACTCCACGCATAACCGCCCAGATAGCCTGAACGATCGACAGTGAATCACCCTCAGTTATGCGTGCGACGGTGAA	−33.00
DDT_26	TCCAGCACTCCACGCATAACCCGCAAGGTGGGTCGGACCTTTCCCCGCTTATACAGACGTCGTTATGCGTGCGACGGTGAA	−34.97
DDT_27	TCCAGCACTCCACGCATAACGAAGACATGCCCGCCAATGGATGTTGTTGGACCTGTTTGAGTTATGCGTGCGACGGTGAA	−35.66
DDT_28	TCCAGCACTCCACGCATAACCGATCGCACCACTCGTGTCAATTGGATTCCACTAGGCCCCGGTTATGCGTGCGACGGTGAA	−39.08
DDT_34	TCCAGCACTCCACGCATAACCCAGGACCGACCCCGCAGACTACAAAGTGTCACTAAGCAATGTTATGCGTGCGACGGTGAA	−31.67
DDT_35	TCCAGCACTCCACGCATAACCCCACAATCCAAGCCGGTCCATCAATGCGGTTATCCATCCTGTTATGCGTGCGACGGTGAA	−31.60

Notes: Sequence with under lines were primer binding sites; Sequences marked with Red color were selected for further analysis.
